# Protective Mechanism of *Acacia saligna* Butanol Extract and Its Nano-Formulations against Ulcerative Colitis in Rats as Revealed via Biochemical and Metabolomic Assays

**DOI:** 10.3390/biology9080195

**Published:** 2020-07-30

**Authors:** Heba M.I. Abdallah, Naglaa M. Ammar, Mohamed F. Abdelhameed, Abd El-Nasser G. El Gendy, Tamer I. M. Ragab, Ahmed M. Abd-ElGawad, Mohamed A. Farag, Mona S. Alwahibi, Abdelsamed I. Elshamy

**Affiliations:** 1 Pharmacology Department, Medical Research Division, National Research Centre, Dokki, Giza 12622, Egypt; fayed.nrc@gmail.com; 2 Therapeutic Chemistry Department, National Research Centre, Dokki, Giza 12622, Egypt; naglaaammar@yahoo.com; 3 Medicinal and Aromatic Plants Research Department, National Research Center, Dokki, Giza 12622, Egypt; aggundy_5@yahoo.com; 4 Chemistry of Natural and Microbial Products Department, National Research Centre, Dokki, Giza 12622, Egypt; tamerragab2006@gmail.com; 5 Plant Production Department, College of Food & Agriculture Sciences, King Saud University, P.O. Box 2460, Riyadh 11451, Saudi Arabia; 6 Department of Botany, Faculty of Science, Mansoura University, Mansoura 35516, Egypt; 7 Pharmacognosy Department, College of Pharmacy, Cairo University, Kasr el Aini St., P.B., Cairo 11562, Egypt; mfarag73@yahoo.com; 8 Chemistry Department, School of Sciences & Engineering, The American University in Cairo, New Cairo 11835, Egypt; 9 Department of Botany and Microbiology, College of Science, King Saud University, Riyadh 11451, Saudi Arabia; malwahibi@ksu.edu.sa; 10 Department of Natural Compounds Chemistry, National Research Center, Dokki, Giza 12622, Egypt; 11 Faculty of Pharmaceutical Sciences, Tokushima Bunri University, Yamashiro-cho, Tokushima 770-8514, Japan

**Keywords:** *Acacia saligna*, ulcerative colitis, nano-extract, inflammation, metabolomics

## Abstract

Ulcerative colitis (UC) is a relapsing inflammatory disease of unknown etiology. The increased risk of cancer in UC patients warrants for the development of novel drug treatments. Herein, this work concerns with the investigation of the protective effects of *Acacia saligna* butanol extract (ASBE) and its nanoformulations on UC in a rat model and its underlying mechanism. Colitis was induced by slow intrarectal infusion of 2 mL of 4% (v/v in 0.9% saline) acetic acid. Colon samples were evaluated macroscopically, microscopically, and assayed for pro-inflammatory cytokine levels. To monitor associated metabolic changes in acetic acid-induced UC model, serum samples were analyzed for primary metabolites using GC–MS followed by multivariate data analyses. Treatment with ASBE attenuated acetic acid-induced UC as revealed by reduction of colon weight, ulcer area, and ulcer index. ASBE treatment also reduced Cyclooxygenase-2 (COX-2), Prostaglandin E2 (PGE2) & Interleukin-1*β* (IL-1*β*) levels in the inflamed colon. The nano-formulation of ASBE showed better protection than the crude extract against ulcer indices, increased PGE2 production, and histopathological alterations such as intestinal mucosal lesions and inflammatory infiltration. Distinct metabolite changes were recorded in colitis rats including a decrease in oleamide and arachidonic acid along with increased levels of lactic acid, fructose, and pyroglutamic acid. Treatment with nano extract restored metabolite levels to normal and suggests that cytokine levels were regulated by nano extract in UC. Conclusion: ASBE nano extract mitigated against acetic acid-induced colitis in rats, and the underlying mechanism could be attributed to the modulatory effects of ASBE on the inflammatory cascades. The applicability of metabolomics developed in this rat model seems to be crucial for evaluating the anti-inflammatory mechanisms of new therapeutics for acute colitis.

## 1. Introduction

Inflammatory bowel diseases (IBD), including ulcerative colitis (UC) and Crohn’s disease, are up to now of unidentified etiology [1]. In fact, its pathogenesis is contributed by multifactorial agents (genetic, immune, and environmental factors). Inflammatory bowel disease, in acute or chronic form, interferes with lifestyle causing a number of health problems for people in both developing and developed countries [2,3]. Rectal bleeding and diarrhea are major symptoms of intestinal enteritis and accounts for about 1.7 billion of reported cases each year [4]. Gastroenteritis is the second leading cause of death globally and accounts for over one million deaths annually [5], and with higher mortality rates in developing countries.

Corticosteroids and 5-aminosalicylic acid compounds are commonly used for the treatment of IBD. However, the side effects of dose-dependent are directly associated with the using of these types of medicinal drugs such as azathioprine and cyclosporine A [6]. Nanocrystalline silver has been shown to exhibit anti-inflammatory properties [7]. In animal models of inflammatory skin disease, NPI 32,101 was shown to decrease inflammation and down-regulates the production of interleukin-12 (IL-12), tumor necrosis factor-alpha (TNF-α) involved in the pathogenesis of inflammatory bowel disease [8].

Several side effects accompanyed conventional treatments for IBD may be mild such as nausea, vomiting, diarrhea, headache, and osteoporosis [8]. In severe cases, patients may experience drug resistance, recurrence, and new IBD symptoms warranting the development of new drugs to treat IBD safely and effectively. To determine the efficacy and safety of drugs, monitoring of global metabolites changes can provide the perfect tool to address such issues in response to novel drugs. Metabolomics is the systematic analysis of metabolites in a biological sample, involving biochemical compounds of low molecular weight, such as organic acids, amino acids, sugars, lipids, and nucleotides [9] using mostly chromatographic techniques coupled to mass spectroscopy. Metabolomics was used to detect and diagnose disease progression early and offered prognostic biomarkers as new therapeutic targets [10]. Mass spectrometry (MS) and nuclear magnetic resonance (NMR) are generally utilized in the metabolomics study [11]. Among these, gas chromatography coupled with mass spectrometry (GC/MS) is commonly used for metabolites profiling in different biofluids, bettering other technical platforms attributable to its high sensitivity and reproducibility, as well as the availability of spectral libraries for structural identification [12].

Recently, the use of herbal drugs or natural products as therapeutic agents has gained much attention worldwide for the treatment of several diseases [13]. Phenolic compounds including flavonoids represent the main components of almost all of the herbal plants [14]. Phenolic compounds including flavonoids and phenolic acids are potent bioactive materials as antiulcer, antioxidant, anti-inflammatory, cardioprotective, anticancer, hepatoprotective, antimicrobial, anti-diabetic agents [14,15].

Plants belonging to *Acacia* genus (Fabaceae Family) are rich in phenolic compounds, flavonoids, and tannins and to mediate for the treatment of haemorrhoids, perspiring feet, diarrhea, skin problems, wounds, eye problems, mouth wash, diabetes, irritable bowel syndrome, and obesity [16]. Several metabolites were reported from *Acacia* species especially flavonoids, cyclitols, tannins alkaloids, saponins, di- and triterpenes cyanogenic glycosides, and essential oils [17]. From the different extracts of *A. saligna*, flavonoids, spirostane saponin, and lignans [18], were characterized with potential antimicrobial [19] and allelopathic activity [20]. Several reports described the role of *Acacia* plants in the management of inflammations and pains in traditional medicines especially in Africa [21]. From this overview, many publications described the biological activities of these plants as anti-inflammatory agents such as *A. modesta* [22], *A. mearnsii* [23], and *A. hydaspica* [24].

Attempts to improve natural products or drug extracts delivery or pharmacological actions has been on going to include among other techniques encapsulation or micro sizing [25]. The scientists made focused strongly on the use of nanoparticles in drug delivery with a 10 to 1000 nm size ranging that have significant enhancements than those of fine and/or bulk particles [26]. The enhancements include improvement of (i) bioavailability via enhancing the solubility, (ii) the time of residence in the body, (iii) the half-life of the clearance and specificity for the cognate receptors, (iv) the targeting of the specific cellular goals, (v) the safety of the drugs via lowering the used drug dose, (vi) the care of non-targeted tissues from serious side effects [27].

With regards to assessment of drug efficacy against UC, rats have a relatively larger body size than mice and in turn, a large intestinal tract which allows for better examination, more tissues to be harvested, and overall more resembles intestinal inflammation in human [28]. Acetic acid-induced colitis is a valid animal model that induces acute inflammatory responses mimicking that seen in UC [29].

This study aimed to enhance the pharmacological activities of ASBE by using nanoparticles delivery system with regard to its anti-UC effect. To achieve such a goal, anti-ulcer effects of ASBE and its nano-extracts were assessed compared to standard anti-inflammatory ‘’dexamethasone’’ using acetic acid-induced UC rat model via monitoring histopathological changes, biochemical markers, and serum metabolites and analysed using chemometrics.

## 2. Materials and Methods

### 2.1. Plant Material, Extract Preparation and Phenolics Characterization

A composite sample from the shoots of *A. saligna* was collected near Gamasa city, north Nile Delta, 31.4293955 N 31.5280741 E, Egypt, in April 2016 and identified according to [30,31]. A voucher specimen (Mans.0060119013) was deposited at the herbarium of Mansoura University, Mansoura, Egypt. The collected sample was cleaned of dust and dried in shade conditions at room temperature (25 ± 3 °C) until complete dryness. The air-dried powder of *A. saligna* shoots (650 g) were macerated in *n*-BuOH at room temperature, filtered, and dried under vacuum to yield dark black residue (26.0 g). A small amount of the *n*-BuOH crude extract (ASBE, 11 g) was stored in sealed air-tight glass vials at 4 °C until further assays.

Total polyphenols content, including total polyphenols (TPC) and total flavonoid contents (TFC), were determined according the previous described protocol [32,33]. The remain *n*-BuOH extract (15 g) was subjected to further fractionation over polyamide 6 L CC, eluted with a mixture of H_2_O/MeOH step gradient and afforded four main fractions (AS-1: AS-4) after final collection according to paper chromatography and TLC profile. Depending upon the Sephadex LH-20 CC and RP-18 HPLC followed by spectroscopic analysis (UV, NMR, and MS), fifteen compounds **1-15** were characterized.

### 2.2. Drugs and Chemicals

Dexamethasone was purchased from Merck Serono, Galaxo SmithKline, UK. Glacial acetic acid, methoxyamine hydrochloride, pyridine, *n*-methyl-*n*-(trimethylsilyl) trifluoroacetamide (MSTFA), trimethylchlorosilane, acetonitrile (99.8%) and *n*-alkane C8-C40 standards were purchased from Sigma-Aldrich (St. Louis, MO, USA). Silver nitrate (AgNO_3_) was purchased from Merck (Burlington, VT, USA).

### 2.3. Biosynthesis of ASBE Silver Nanoparticles (Ag-NPs)

Ag-NPs were prepared by the reduction of AgNO_3_ using ASBE. Stock aqueous 10 mL AgNO_3_ solution (1 mM) was added at different volumes (100, 200, 300, 400 and 500 µL) of the extract at 25 °C. The reaction mixture was shaken slightly and allowed to stand in the dark overnight [34]. The Ag-NPs were prepared through reducing silver nitrate (AgNO_3_) with ethylene glycol (EG) in the existence of polyol. In a classic method, 10 mL of EG was refluxed at 160 °C for 25 min. Then, 5 mL solution of AgNO_3_ in EG and 10 mL solution of 0.15 M PVP in EG containing 0.03 mM MnCl_2_ was concurrently be injected into the flask over a period of 10 min, the reaction mixture was further refluxed and vigorously stirred at 160 °C for 60 min. The reaction mixture was then cooled to room temperature followed by centrifugation at 3000 rpm for 5 min, and then washed with acetone and ethanol three times. The Ag-NPs were collected and dried in an oven at 70 °C. Nanoparticles are defined as particulate dispersions or solid particles with a size in the range of 1–100 nm. The emulsion process of plant extract nanoemulsion was prepared using plant extract, non-ionic surfactant Tween 20 (HLB-16.7), and water via spontaneous emulsification method. Nanoemulsion was carried out in two steps: First, the organic phase was fabricated through mixing plant extract sample with chosen surfactant (Tween 20) in the following ratio (1:5). In the second step, the organic phase (a plant extract and Tween 20) was added drop by drop (20 mL/min) to water using separating funnel and stirring the system magnetically (800 rpm). Then the prepared silver nanoparticles (Ag-NPs) are added to the prepared nanoemulsion by the following ratio (1%) followed by sonication for another 30 min at 50 °C.

### 2.4. UV–vis Spectra Measurements

The absorbance spectrum of produced ASBE nanoparticles was measured immediately (within 1 h) after its synthesis. Before recording the absorbance spectrum, a reference of de-ionized water was recorded, as appropriate. The spectrophotometer used was a UV-2401 (PC)S, UV-Visable recording spectrophotometer (Shimadzu, Japan) and the software was Spectrum™ Version 6.87. Absorbance spectra were calculated over the range of 200–800 nm. The wavelength of peak absorbance (λ_max_) was noted.

### 2.5. Transmission Electron Microscopy (TEM)

Particle size and shape were determined with a JEOL JEM 1011 (Japan) transmission electron microscope. 400 μL of nanoparticle solution was deposited on carbon-coated copper grids (400 mesh) and dried at 30 °C before image capture.

### 2.6. Experimental Design

Animals were randomly allocated into eight groups, each one with six animals. Group 1: negative control (treated with saline). Group 2: positive control group (receiving acetic acid as mentioned above). Group 3: reference group (receiving dexamethasone intraperitoneally at a dose of 1 mg/kg) (Dolatabadi and Zarchii, 2015). Group 4: each rat received 2 mL of nano-Ag (Ag) solution (100 mg/L). Group 5: received ASBE at a dose of 100 mg/kg as reported in previous literature [35]. Group 6: received ASBE and nano-Ag. Group 7: received nanoformulation of ASBE at the same dose (100 mg/kg). Group 8: received nanoformulation of ASBE and nano-Ag. All groups except the negative control group received acetic acid (as mentioned above). The extract, its nanoformulation, dexamethasone, or nano-Ag were administered orally administered daily for 2 weeks before starting UC induction with acetic acid and continued for further 2 days.

### 2.7. Blood Collection, Colon Lesions Assessment, and Tissue Preparation

After 48 h of acetic acid injection, blood samples were collected from the retro-orbital venous plexus of rats under light ether anesthesia and collected in clean test tubes, left to clot, then centrifuged for 10 min at 3000 rpm using a cooling centrifuge (Sigma and Laborzentrifugen, 2 k15, Germany). Serum was separated and stored into Eppendorf tubes at −80 °C to be used for metabolomics study.

After the collection of blood samples, animals were sacrificed by cervical dislocation, and laparotomy was performed. Colonic segments (8 cm in length and 3 cm proximal to the anus) were rapidly excised, gently cleaned of fecal contents, and opened along the mesenteric border, washed with saline. Colon specimen wet weight/length (g/cm) ratios were calculated and visually inspected for ulcerogenic features. Mucosal lesions were also quantified by a [36] 0–5 scoring system with 0 for no damage, 1 for localized hyperemia, 2 for ulcers or erosions with no significant inflammation, 3 for ulcers or erosions with inflammation at one site, 4 for two or more ulceration sites and/or inflammation, and 5 for two or more major sites of inflammation and ulceration or one major site of inflammation and ulceration extending more than 1 cm along the length of the colon. Ulcer area was measured using a plane glass square. Each cell on the glass square was 1 mm^2^ in area, the number of cells was counted and with ulcer area was determined for each colon. Ulcer index was measured by summing the lesion score and the ulcer area for each colon specimen [37].

Colons of rats were divided into two parts, the first colon samples were homogenized and with the homogenate kept at (−80 °C) for biochemical determination of colon content of cyclooxygenase 2 (COX-2), prostaglandin E2 (PGE2), and interleukin 1 beta (IL1β). The second colon sample was preserved in phosphate-buffered formalin 10% for histopathological investigation.

### 2.8. Metabolomics Samples Preparation and GC–MS Analysis

Samples were randomized prior to sample preparation. Serum samples were thawed on ice and with metabolites extracted as described by Hassan, et al. [38] as follows: 100 μL of serum was mixed with 200 μL of cold acetonitrile (100%) containing 5 µg xylitol as an internal standard, centrifuged at 7000 rpm for 15 min followed by centrifugation. The supernatant was evaporated using nitrogen gas and a freeze dryer till complete dryness. For metabolites derivatization, a volume of 50 μL methoxyamine HCl/pyridine solution (20 mg/mL) was first added to the dried pellet then incubated at 60 °C for 1 h. As a second step derivatization, 100 μL MSTFA containing 1% TMS was added to the mixture then incubated at 60 °C for 1 h. The quality control (QC) sample was prepared by mixing aliquots of all samples to be a pooled sample.

### 2.9. GC–MS Analysis

Gas Chromatography (Thermo Scientific Corp, USA), coupled with a thermo mass spectrometer detector (ISQ Single Quadrupole Mass Spectrometer) was used for the profiling of serum metabolites. Chromatographic separation was achieved as described by Raish, et al. [39] with some modifications, using TG-5MS column (30 m × 0.25 mm i.d., 0.25 μm film thickness) and helium as carrier gas at a flow rate of 1.0 mL/min with a split ratio of 1:10 using the following temperature program: 80 °C for 2 min; rising at 5.0 °C/min to 300 °C and held for 5 min. The injector and detector were held at 280 °C. Mass spectra were obtained by electron ionization (EI) at 70 eV, using a spectral range of m/z 35–500.

### 2.10. GC-MS Data Processing and Multivariate Chemometric Analysis

Serum metabolites were identified by comparing their retention indices (RI) relative to *n*-alkanes (C8–C40) standards along with mass matching to NIST library database. Peaks were first deconvoluted using AMDIS software (www.amdis.net) prior to mass spectral matching. Metabolites abundance data were prepared for multivariate data analysis by extraction using MET-IDEA software for data extraction. The MS signals abundance were normalized to the internal standard “xylitol” in each sample and further subjected to Pareto scaling prior to multivariate data analysis. Data were then subjected to principal component analysis (PCA), orthogonal projection to latent structure discriminate analysis (OPLS-DA) using SIMCA-P software.

### 2.11. Biochemical Assessment of Colon Tissue

Cyclooxygenase 2 (COX-2), prostaglandin E2 (PGE2), and interleukin 1 beta (IL-1*β*) levels were determined in intestinal homogenate using ELISA available kits following the manufacturer’s instructions (Glory science, Texas, USA).

### 2.12. Histopathological Investigation

Tissue specimens from colon were processed as follows: dehydrated, embedded in paraffin, sectioned at 3–5 µm thickness, and finally stained with hematoxylin-eosin stain (H&E) for the determination of histopathological changes that occurred in colon tissue. The sections were scanned and analyzed by a pathologist not aware of sample assignment to experimental groups [40].

### 2.13. Statistical Analysis

All results were expressed as mean ± standard error of the mean. Data analysis was achieved by one-way analysis of variance (ANOVA) followed by Tukey comparison test using software program GraphPad Prism (version 6.00). The difference was considered significant when *p* < 0.05. Univariate analysis was performed using SPSS version (16) for the biochemical data analysis. Nonparametric (Mann-Whitney test) was used to compare between two groups after normalization with a kruskal-Wallis test.

## 3. Results and Discussion

### 3.1. Chemical Characterization of ASBE

The abundance of polyphenolic compounds in *Acacia* plants and especially *A. saligna* was previously reported [18,19] posing it to be examined in this study for its anti-inflammatory effects. The results of polyphenolic determination in ASBE revealed the abundance of total phenolic and flavonoid contents at 68.39 mg/g extract and 143.84 mg/g extract, respectively. The chemical characterization via different chromatographic techniques further identified 15 phenolic compounds including six flavonoids, naringenin (1), taxifolin (2), catechin (3), quercetin (4), rutin (5), and kaempferol (6), and nine phenolic compounds, gallic acid (7), methyl gallate (8), syringic acid (9), cinnamic acid (10), ferulic acid (11), coumaric acid (12), caffeic acid (13), ellagic acid (14), and chlorogenic acid (15) (Figure S1).

### 3.2. Characterization of ASBE Silver Nanoparticles (Ag-NPs)

ASBE-Ag-NPs physicochemical properties were initially characterized to ensure its biodistribution, safety, and effectiveness. The characterization of Ag-NPs was based on its UV characterization and transmission electron microscopy (TEM) to determine the functional aspects of the synthesized particles.

### 3.3. UV-vis Spectroscopy

**Figure 1:**
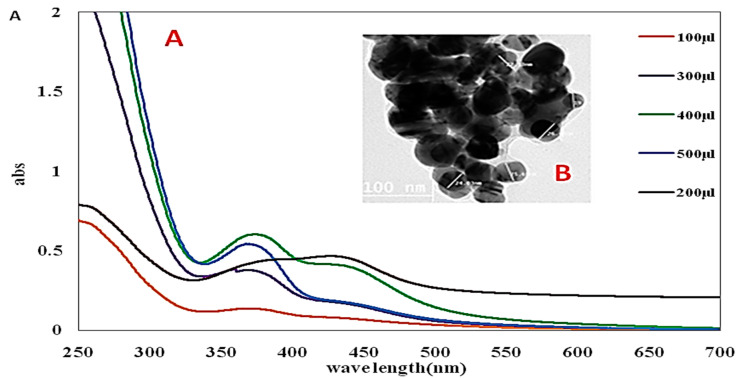
(**A**) The SPR band of Ag-NPs recorded by UV–vis spectra as a function of varying concentrations (100, 200, 300, 400, and 500 µL). (**B**) TEM image of A ASBE-Ag-NPs.

UV-vis spectroscopy is a common tool for the characterization of synthesized nanoparticles, also used to examine Ag-NPs synthesis and stability (Sastry et al., 1998). In Ag-NPs, the transmission and valence band lie very close to each other in which electrons move freely. The absorption of Ag-NPs depends on the size of particles, dielectric medium, and chemical environment. ASBE-Ag-NPs solution color was wide-ranging from yellow/colorless to dark brown depending upon the concentration of the formed nanoparticles. No remarkable aggregation of particles and color-changing were detected even after 30 min. The aggregation was observed via the color alteration to darkness and then colorless solutions with the formation of a black precipitate of silver. At different concentrations, characteristic UV-vis spectra of silver nanosized particles at the plasmon band between 445–456 nm [41]. The data of Figure 1A revealed an absorption band appeared at longer wavelengths with small spherical Ag-NPs. The surface plasmon resonance (SPR) band intensity increased with concentration and that more Ag^+^ ions are reduced to Ag nanoparticles [34].

### 3.4. Transmission Electron Microscopy (TEM)

TEM is a versatile technique for nanomaterials characterization, used to obtain quantitative measurements of particle and/or grain size, size distribution, and morphology (Lin et al., 2014). High-resolution transmission electron microscopy (HRTEM) technique was used for evaluation of the size and shape of the designed Ag-NPs. The TEM image of the prepared Ag-NPs distribution was presented in Figure 1B. The ASBE-Ag-NPs were characterized by spherical shapes along with particle size range 20 to 40 nm. The particles were separable from each other to reflect the action of capping of the extract during the process of preparation. The integration of ASBE-Ag-NPs was formed with a particle size of 15–56 nm [42]. Due to their unique properties, ASBE-Ag-NPs was ready for extensively evaluation for UC in rats.

### 3.5. Effect of ASBE and Its Nanoformulations against Acetic Acid induced UC in Rats Model Effect on Macroscopic Features

**Figure 2:**
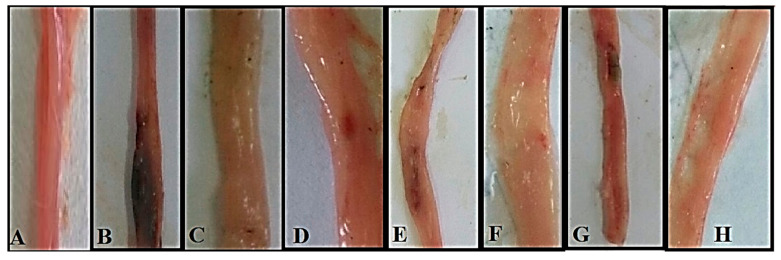
Photograph of Macroscopic features of rat colon shows (**A**) Negative control: normal colon tissue with no apparent damage. (**B**) Positive control: considerable inflammation, ulceration, wall thickening, necrosis and gangrenous changes in colon tissue (**C**) Dexamethasone standard: apparent normal with no damage in colon tissue. (**D**) Nano-Ag: mild inflammatory changes, no evidence of ulceration showing significant improvement (**E**) ASBE: moderate inflammatory changes & signs of ulceration showing little improvement (**F**) ASBE plus nano-Ag: mild inflammatory changes, no evidence of ulceration showing significant improvement (**G**) ASB nano-extract: moderate edematous changes, moderate signs of ulceration showing little improvement (**H**) ASB nano extract plus nano-Ag: apparent normal no evidence of damage in colon tissue.

Macroscopic features of colon tissue (negative control) revealed normal intact tissue with no signs of inflammation or damage (Figure 2A), while in the positive control group, the colon tissue showed typical signs of advanced inflammation, ulceration, wall thickening, necrosis and gangrenous changes in colon tissue (Figure 2B). The reference group which received (1 mg/kg, i.p) dexamethasone as positive drug control showed apparent normal with no damage in colon tissue (Figure 2C). The little improvement observed by treating ASBE or ASB nano extract were images of colon tissue revealed moderate inflammatory changes and some signs of ulceration (Figure 2E,G). Albeit the edematous changes are more prominent in the colon of the ASB nano extract-treated group (Figure 2G). The combination therapy seems to be thus the best therapeutic strategies where the ASBE combined with nano-Ag group showed the most improvement, with just mild inflammatory changes and no evidence of ulceration, necrosis nor gangrenous changes (Figure 2F). Rat group treated with ASB nano extract combined with nano-Ag (Figure 2H) represented the best protocol in the combination therapy where its colon tissue showed apparent normal, no evidence of any damage seems to be mimic to negative control. This synergistic effect is supported by the observation that colon treated with the nano-Ag vehicle only improved the ulceration point but still not protect against acetic acid inflammatory actions (Figure 2D).

### 3.6. Effect on Colon Ulcer Indices

Intestinal ulceration was triggered by acetic acid as evidenced by the significant increase in wet weight/length ratio of colon specimens demonstrating the presence of inflammation (Table 1). The wet weight/length ratio increased by about 2-fold in rats with acetic acid colitis (control positive) compared to negative control rats (0.251 versus 0.127 g/cm; respectively). This ratio was reduced in groups pre-treated with ASBE, nano-extract and the combination of either the crude extract or the nano-extract with nano-Ag (0.202 ± 0.007, 0.193 ± 0.007, 0.174 ± 0.006 and 0.146 ± 0.005 g/cm; respectively) as well as dexamethasone (standard anti-inflammatory drug) (0.155 ± 0.007 g/cm). Groups treated with dexamethasone, nano-Ag, ASB nano ext. + nano Ag showed wet weight/length ratio that was not significantly different from control negative group.

**Figure 3:**
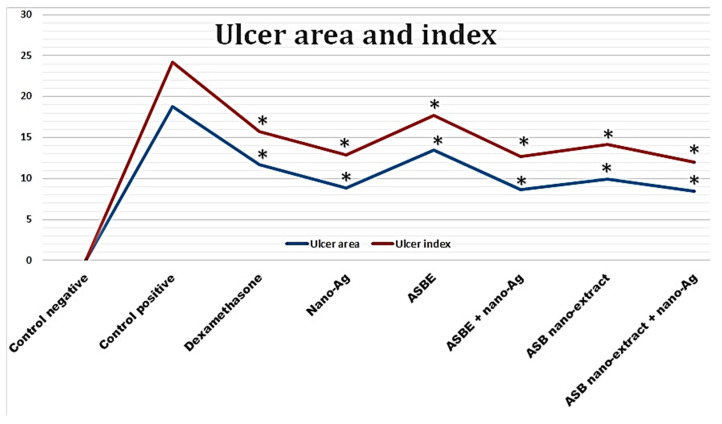
Effect of ASBE and its nano-extracts and their combination with nano-Ag on colon ulcer indices. Results are presented as mean ± S.E.M (n = 6). Statistical analysis was performed using one-way ANOVA followed by Tukey test. * Statistically significant from control positive at *p* < 0.05.

Similar to increased wet weight/length ratio, positive control colitis rats exhibited elevated values of lesion scores, ulcer areas, and ulcer index values (5.50 ± 4.39, 18.75 ± 0.49 mm^2^, and 24.25 ± 0.77; respectively) (Table 1 and Figure 3). These inflammatory changes were significantly reduced by ASBE, nano extracts, and their combination with nano-Ag. The combination of nano-extract with nano-Ag afforded the greatest protection against increased ulcer index induced by acetic acid to reach 50% compared with 35% for dexamethasone and 27%, 42%, 48%, and 47% for ASBE, ASB nano-extract, ASBE plus nano-Ag, and nano-Ag. Control positive, dexamethasone, ASPE, ASB nano ext. groups showed only 16.66% mortality. However, other groups did not show any mortality.

### 3.7. Effect on Inflammatory Biomarkers (COX-2, PGE_2_, IL-1β)

**Figure 4:**
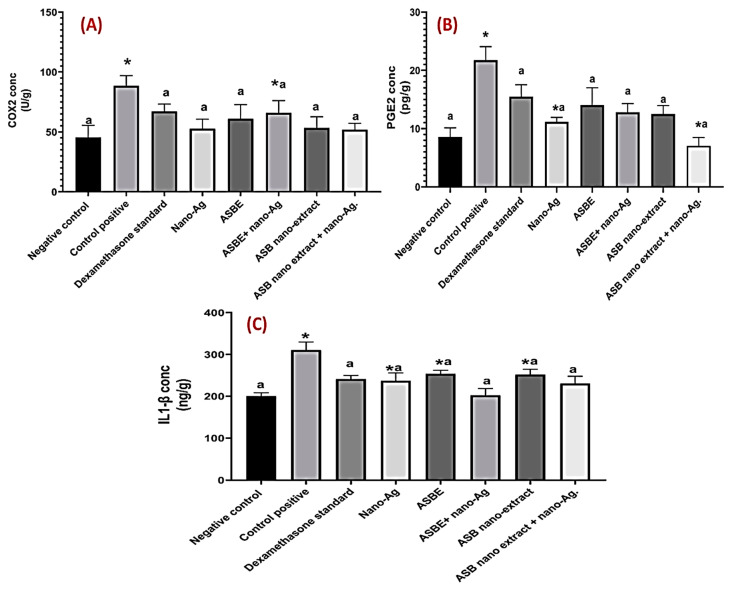
Effect of ASBE and nano-extracts and their combination with nano-Ag on (**A**) COX2 content in colon tissue, (**B**) on PGE2 content in colon tissue, and (**C**) IL-1β content in colon tissue. Results are presented as mean ± SD (n = 6). Statistical analysis was performed using one-way ANOVA followed by Tukey test. * Statistically significant from control negative, a Statistically significant from control positive at *p* < 0.05.

Injection of acetic acid to rats resulted in increased activity of colon COX-2 as compared to the negative control group. However, administration of dexamethasone, crude ext., nano ext., crude ext. + nano-Ag, nano ext. + nano-Ag, and nano-Ag significantly (*p* < 0.05) decreased COX-2 as compared to acetic acid positive control group. The suppression of COX-2 with different treatments was comparable to the reference drug, dexamethasone. No significant difference was observed between all treatment groups (Figure 4A).

Consequently, acetic acid injection markedly increased the production of the inflammatory mediator, PGE2 by about 2.5 folds as compared to the negative control group. This finding is probably due to increased synthesis by the action of COX-2 enzyme. All treatments induced a significant decrease in the colon content of PGE2 as compared to an acetic acid group. However, nano ext. + nano-Ag and nano-Ag groups; respectively showed the highest impairment of PGE2 production at ca. 68%, and 49%; respectively vs. 29% for dexamethasone as compared to acetic acid group (Figure 4B). Nano extract decreased PGE2 production by only 21%.

Finally, IL-1*β* content was induced in rats colon after receiving acetic acid only as compared to the negative control group. Treatment with dexamethasone, nano ext., crude ext. plus nano-Ag, nano extract plus nano-Ag, and nano-Ag decreased in a statistically significant manner (*p* < 0.05) IL-1β as compared to acetic acid positive control group. IL-1*β* levels in these groups were not significantly (*p* < 0.05) different from that of dexamethasone (Figure 4C).

### 3.8. Histopathological Changes

**Figure 5:**
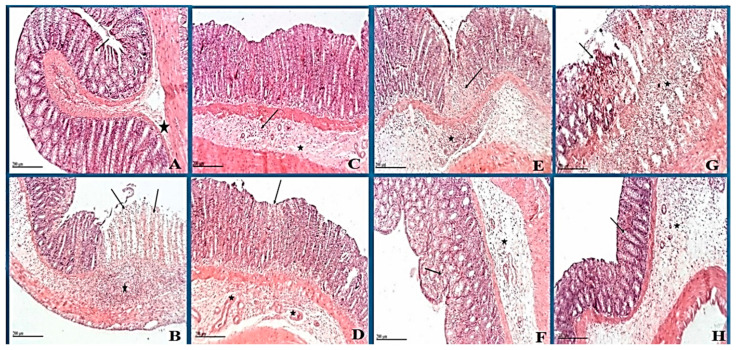
Photomicrograph of rat colon shows (**A**) negative control: normal histological structures of the colon with intact mucosa (star) including intestinal crypt with abundant goblet cells (arrow). (**B**) Positive control: extensive focal erosions and ulceration (arrow) of intestinal mucosa with necrotic tissue debris. Extensive inflammatory cells infiltration including mononuclear and polymorphonuclear cells in mucosa and submucosal layers (star) accompanied with moderate submucosal edema. (**C**) Dexamethasone standard: Apparent intact glandular mucosa and lining epithelium with moderate inflammatory cells infiltration in deeper mucosa and submucosal layer (arrow) accompanied with mild submucosal edema (star). (**D**) Nano-Ag: few focal areas of necrosis in the superficial zone of intestinal mucosa (arrow) with many intact glandular elements. Mild submucosal edema and inflammatory cells records with many dilated and/or congested submucosal blood vessels (star). (**E**) ASBE: tiny focal erosions and ulceration (arrow) of intestinal mucosa with necrotic tissue debris. Focal inflammatory cells infiltration including mononuclear and polymorphonuclear cells in mucosa and submucosal layers (star) accompanied with mild submucosal edema. (**F**) ASBE plus nano-Ag: apparent intact mucosa including glandular structures with many goblet cells. Moderate inflammatory cells infiltration in interglandular tissue (arrow) as well as submucosal layer (star) with diffuse submucosal edema. (**G**) ASB nano-extract: erosions and ulceration (arrow) of intestinal mucosa with necrotic tissue debris. Inflammatory cells infiltration including mononuclear and polymorphonuclear cells in mucosa and submucosal layers (star) accompanied with mild submucosal edema. (**H**) ASB nano extract plus nano-Ag: apparent intact mucosa including glandular structures with many goblet cells(arrow) with fewer inflammatory cells infiltration records. Focal areas of submucosal edema (star) (Normal submucosa). (H&E stain, 100 X); Scale bars for all figures (**A**–**H**) are 20 µm.

Histopathological examination of colon specimen negative control showed normal histological structures with intact mucosa including intestinal crypt with abundant goblet cells, intact submucosa and muscular layers (Figure 5A), while in the positive control group, the microscopic examination showed extensive focal erosions and ulceration of intestinal mucosa with necrotic tissue debris that occasionally extend to muscular coat with extensive inflammatory cells infiltration. Mononuclear and polymorphonuclear cells were recorded in the mucosa and submucosal layers accompanied by moderate submucosal edema (Figure 5B). Reference group which received (1 mg/kg, i.p) dexamethasone as positive drug control showed apparent intact glandular mucosa and lining epithelium, albeit with still moderate inflammatory cells infiltration in deeper mucosa and submucosal layer accompanied with mild submucosal edema (Figure 5C). Groups treated with nano-Ag showed few focal areas of necrosis of intestinal mucosa with many intact glandular elements; mild submucosal edema and inflammatory cells with many dilated and/or congested submucosal blood vessels was observed (Figure 5D). Microscopic picture revealed little improvement in ASBE treated group (100 mg/kg), with intestinal mucosa showing tiny focal erosions and ulceration with necrotic tissue debris that occasionally extend to muscular coat and focal inflammatory cells infiltration including mononuclear and polymorphonuclear cells accompanied with mild submucosal edema (Figure 5E).

Likewise, ASB nano extract treated rats (Figure 5G) showed little improvement. Erosions and ulceration of intestinal mucosa with necrotic tissue debris were observed and occasionally extended to a muscular coat. Inflammatory cells infiltration including mononuclear and polymorphonuclear cells was also observed in the mucosa and submucosal layers accompanied by mild submucosal edema. Histopathological examination of the ASBE combined with the nano-Ag group that showed the most improvement, with apparent intact mucosa including glandular structures with many goblet cells (Figure 5F). Nevertheless, moderate inflammatory cells infiltration was recorded in interglandular tissue as well as submucosal layer with diffuse submucosal edema. Rat group treated with ASB nano extract combined with nano-Ag (Figure 5H) showed apparent intact mucosa including glandular structures with some goblet cells with fewer inflammatory cells infiltration records and showing the most improved picture close to the negative control. Focal areas of submucosal edema (normal submucosa).

The present investigation outlines the anti-ulcerogenic effect of ASBE and its nano-extract formulation against experimentally induced UC in rats. The protective effect of ASBE and its nano-extract against colon ulcers was confirmed at multiple levels including histological evaluation, biochemical assays, and serum metabolites analysis, by using dexamethasone as a positive standard drug. Forteen days pretreatment with ASBE and its nano-extract significantly reduced acetic acid-induced colonic ulcerative lesions and prevented its inflammatory response.

UC is characterized by mucosal inflammation and ulceration with a variable extent of severity [43]. Rectal administration of 4% acetic acid for the induction of UC in rats is a well-established animal model, which phenotypically resembles colon inflammation in humans [44]. It also mainly causes colonic epithelial lesions and necrosis associated with infiltration of the damaged colon with neutrophils and macrophages indicating inflammatory conditions. In the current study, administration of acetic acid significantly increased colonic weight and induced marked ulceration associated with inflammatory infiltration, necrosis, and goblet cell hyperplasia as revealed from both macroscopic examinations (Figure 3). Similar pathological impairments were reported in earlier studies using the same animal model [45,46]. Application of acetic acid perturbed the colonic mucosa in accordance with Popov, et al. [47]. Although numerous medicaments have been suggested for UC treatment, side effects or toxicity of these medications pose a problem and warranted for the discovery of other safe treatments [48,49]. For example, despite mesalamine is a very safe and effective medication, yet its use is associated with paradoxical diarrhea and interstitial nephritis [50]. Likewise, corticosteroids have many complications that are irreversible such as obesity, hypertension, diabetes, cataracts, glaucoma, depression, anxiety, insomnia, irritability, and avascular necrosis [51,52]. Thiopurines (azathioprine [AZA] and 6-mercaptopurine [6-MP]) have multiple side effects ranging from an elevation of transaminases till serious leukopenia [53]. The current treatment with *Acacia Saligna* herb afforded protection against unpleasant morphological changes induced by acetic acid as well as abnormal ulcer indices (Table 1). Hence, the herb is suggested to be a safe natural therapeutic modality for ulcerative colitis and prevention of its consequent damage though less effective compared to its nanoformulation.

Inflammatory cytokines play a fundamental role in modulating the mucosal immune system. Stimulated neutrophils and macrophages disrupt epithelial integrity and thus leading to colon injury [54]. During the pathogenesis of UC, migration of granulocytes and other leukocytes takes place to the inflamed mucosa and superficial ulcers inducing the production of pro-inflammatory cytokines especially IL-1*β* [55]. In the present study, elevated colon level of PGE2, COX2, and IL-1*β* in acetic acid ulcer group is paralleled with, edema and neutrophil infiltration presented by histopathological examination. These results are in compliance with earlier experimental and clinical studies [56,57]. Colonic tissue inflammatory cytokine levels of interleukins were significantly higher in the acetic acid group than the other groups and to comply with that of Tahan, et al. [56]. Treatment with dexamethasone, crude extract, nano-extract, crude extract + nano-Ag, nano extract + nano-Ag, and nano-Ag decreased IL-1*β* as supported by Bessler*,* et al. [58] and Nehmé and Edelman [59] rationalized via dexamethasone acting as an inhibitor for IL-1*β* [60] and also observed in case of nano-Ag to decrease IL-1*β* (Figure 4).

The observed increased colonic PGE2 content in the control ulcer group is in parallel with Otani, et al. [61]. COX-2 catalyzes oxidative stress-induced production of NO and the inflammatory mediators; prostaglandins that are important in the pathogenesis of colitis [62].

Histopathological examination also confirmed biochemical findings where intra-colonic administration of acetic acid showed extensive focal erosions and ulceration of intestinal mucosa with necrotic tissue depress that occasionally extend to a muscular coat. Extensive inflammatory cells infiltration including mononuclear and polymorphonuclear cells was revealed in the mucosa and submucosal accompanied by moderate submucosal edema [63]. Pre-treatment with ASBE crude and nano-formulations could depress COX-2 activity and PGE2 production showing the anti-inflammatory effect of this natural herb. In addition, it improved histopathological abnormalities regarding ulceration, erosions, and inflammatory infiltration induced by acetic acid (Figure 5).

Being enriched extract in flavonoids and phenolic compounds functioning as antioxidant and anti-inflammatory agents, all identified compounds have the potential for treating ulcers [15]. Further, the loading of bioactive extracts and metabolites in nanoparticles is one of the strategies to enhance its efficacy via increasing metabolites stability and its penetration in the tissues and cellular uptake [64]. In parallel, in the current study, the formulation of ASBE as nanoparticles enhanced its anti-ulcerogenic activity. A critical approach to new forms of treatment of Crohn’s disease and ulcerative colitis sulfasalazine [65]. Another recent report has demonstrated that nano-Ag could alleviate colitis through suppression of neutrophil recruitment, infiltration, and modification of colonic microbiota [66].

### 3.9. GC/MS Metabolomics Analysis of Rat Serum

**Figure 6:**
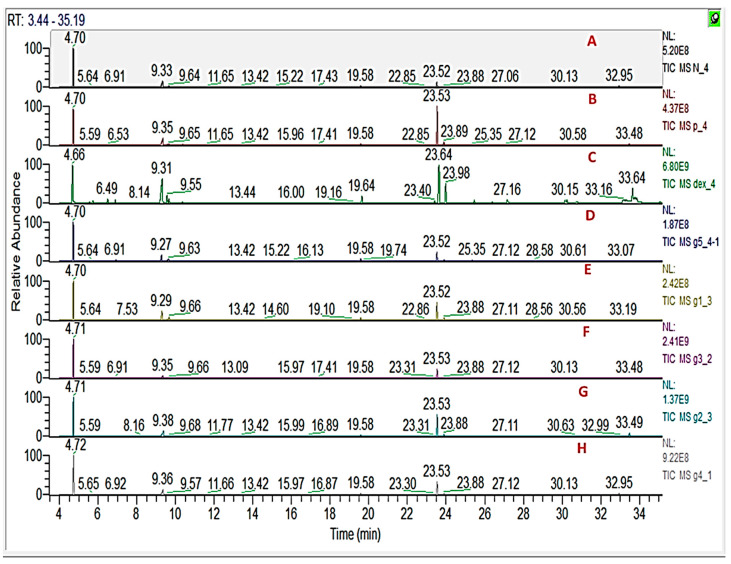
Representative GC/MS chromatograms of serum derived from (**A**) negative control, (**B**) positive control, (**C**) dexamethasone standard, (**D**) nano-Ag, (**E**) ASBE, (**F**) ASBE plus nano-Ag, (**G**) ASB nano-extract, and (**H**) ASB nano extract plus nano-Ag.

To further elucidate underlying metabolite changes accompanying ulcer induction and its prevention using a different treatment, rat serum was analysed for its metabolites profile and analysed using GC/MS. Representative GC/MS total ion current chromatograms from normal control and colitis rat are presented in Figure 6 showing qualitative differences in peak abundance. A total of 44 metabolites were recognized in rat’s serum samples based on RI and matching to spectra in NIST/Wiley databases (Table S1). To assist in identifying metabolite differences and marker for the disease, multivariate data analysis was employed using MS features abundance extracted from the GC/MS. Supervised Orthogonal Projections to Latent Structures Discriminant Analysis (OPLS-DA) was used for rat group classification.

**Figure 7:**
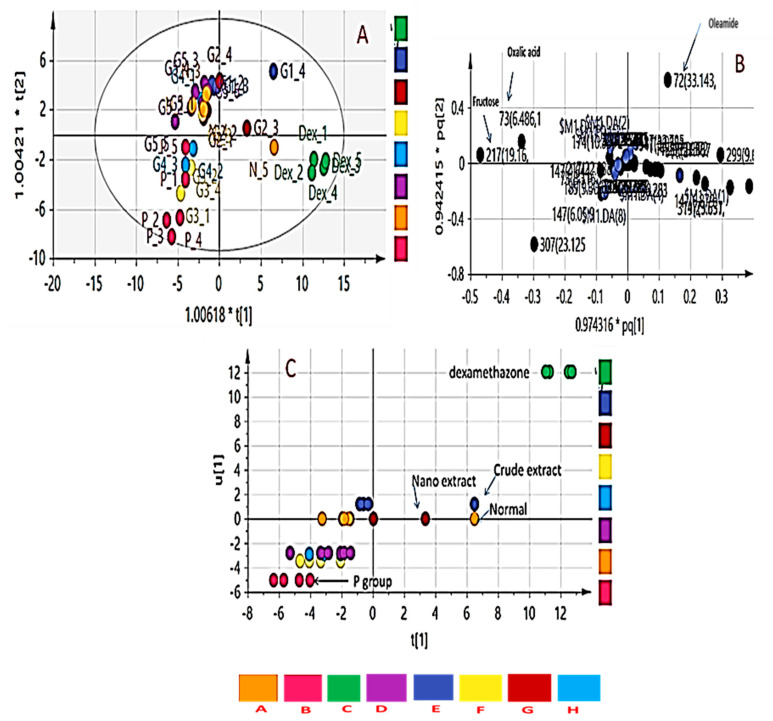
Orthogonal partial least-squares-discriminant analysis (OPLS-DA) score plots obtained from modelling serum metabolites detected using GCMS in the different rat groups. (**A**) Score plot showing a separation of healthy control animals, treated groups and colitis rats, (**B**) Loading plot for PC1 and PC2 components contributing peaks. (**C**) Classes inner relation of the eight rat groups with colitis group showing separation from the normal control group and treatment groups. (**A**) negative control, (**B**) positive control, (**C**) dexamethasone standard, (**D**) nano-Ag, (**E**) ASBE, (**F**) ASBE plus nano-Ag, (**G**) ASB nano-extract, and (**H**) ASB nano extract plus nano-Ag.

OPLS-DA has been used to recognize metabolic trends that are linked with the phenotypic variable of interest while down-weighting the other sources of variance. OPLS-DA score plot (Figure 7A) displayed clear discrimination among sample groups; though with a low R2 value (0.32) and Q2 value (0.13). The loading plot that emerged from OPLS-DA model is represented in Figure 7B showing change levels in metabolites due to colitis induction and or treatment. Metabolites contributing to groups segregation include oleamide, fructose, and oxalic acid.

**Figure 8:**
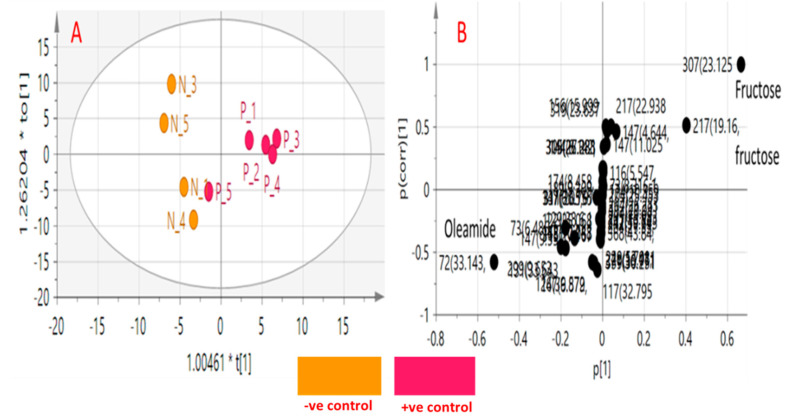
Orthogonal partial least-squares-discriminant analysis (OPLS-DA) score plots obtained from modeling colitis (p) versus healthy control groups (N) against each other. (**A**) Score plot of PC1 and PC2. (**B**) S-plot showing the covariance p [1] against the correlation p(cor) [1] of the variables of the discriminating component. Each metabolite in panel B is denoted by its quantification mass and (peak retention time in min).

OPLS-DA class inner relationship model of all treatment groups is represented in Figure 7C with colitis rats found the most distant from normal rat group with notable differences in their metabolite profiles and in agreement with GC-MS results (Figure 6). Among all studied treated rat groups, nano extract group (G2) was the closest to the normal control group in OPLS-DA plot (Figure 7A), proposing that this group was the most effective to return a metabolite profile in ulcerative colitis rats mirroring that in the normal ones. OPLS-DA model and its derived loading S-plot were further used to pinpoint metabolite markers related to colitis by modeling healthy control versus only colitis rats in a separate model (Figure 8A,B). Improved R2 and Q2 values of normal versus UC rats were noted indicative of covered variance and prediction power for assessing model validity. The first 2 components in OPLS model presented in Figure 8A explained 78% (0.78) of the total variance (R2), with the prediction goodness parameter Q2 = 0.44. R2 did not exceed Q2 by more than 2 units and with no negative Q2 values indicating the validity of the model. Fructose was found most elevated in the rat colitis group, whereas oleamide was noticed at lower levels compared to the healthy control group.

To pinpoint metabolites responsible for the classification, relative quantification of metabolites was performed and statistical analysis was further undertaken. Additionally, relative metabolites fold change was computed across treatment and is presented (Table 2). Consistent with the OPLS-DA derived results (Figure 8), a significant decrease in oleamide and arachidonic acid levels concurrent with an increase in lactic acid, fructose, and pyroglutamic acid in colitis group as compared to healthy rats (Table 2). However, the nano extract group re-normalized the UC-induced metabolic changes in rats.

In the current study, GC-MS was used to analyze rat’s serum metabolic profiles (Table S1) treated with ASB nano-extract to help elucidate its function mechanism of nano-ASBE for treating UC. The UC group was distinguished by higher serum levels of lactic acid, fructose, and pyroglutamic acid, albeit lower levels of arachidonic acid and oleamide compared to the normal control group (*p* < 0.05) (Table 2). OPLS-DA model of derived GC-MS dataset showed a clear separation not only between control and acetic acid-induced colitis groups, but also between acetic acid-induced colitis and dexamethasone treatment groups (Figure 7). These results propose that serum metabolites can provide an indication of colitis induction.

In the present study, UC group showed elevated levels of lactic acid and fructose compared to control and ASB nano-extract treatment groups. More findings have shown that the occurrence of colitis is related to increases in lactic acid levels. For example, Song, et al. [67] reported that lactic acid in the inflammatory intestinal mucosa of IBD patients were reported at higher levels. Another cause of increased lactic acid may be due to changes in the survival of the microorganism in the intestine. Increased concentration of intraluminal oxygen due to excessive bleeding favors facultative anaerobic strains known as lactic acid producers [68]. Enhanced lactic acid concentrations can also result from colonic mucosal cell lining disruption and mesenchymal polysaccharides exposure to intraluminal bacteria [68].

Likewise, increased lactate serum levels are probably secreted from inflamed colonic mucosa cells [69] demonstrating glycolysis up-regulation that occurs in the case of excess cellular energy requirements and/or depletion of energy sources under inflammatory disorders [70]. Upon intervention with ASB nano-extract, a decrease in lactic acid level was observed in treated rats suggesting an improvement in colon function (Figure 7 and Figure 8).

In relation to sugars i.e., monosaccharides, higher levels of fructose and glucose were detected in the UC group compared to the control group [71]. High levels of glucose have been reported in extracts of macroscopically uninvolved colonic mucosa of IBD patients [72], and also in other biological specimens including fecal extracts in patients with ulcerative colitis [73]. In support of such a hypothesis, energy-related metabolites in colon appear to be crucial for homeostasis of the gut microbiome and intestinal cells [74,75], and to suggest that increased glucose level in serum samples of the UC rats may be resulting from the disturbance in the host-microbiome system [76].

Another metabolite that showed the opposite pattern to glucose being detected at lower levels in the UC model is oleamide likely attributed to increased inflammation [77]. Oleamide levels showed significant increase after nano extract administration compared to the untreated colitis group and suggestive for its efficacy at the metabolite level. Oleamide is the amide of oleic acid produced by enzymatic amidation from oleic acid and ammonia [78]. It is a novel metabolite with anti-inflammatory properties, besides a strong dual-active component able to improve both phagocytosis and anti-inflammatory activity [78,79,80], that has yet to be tested for clinically to reveal its role in ulcerative colitis.

A prior study using an LPS-stimulated murine microglial cell line (BV-2) revealed that oleamide inhibited the production of NO and PGE2 [81]. Other metabolites that showed down-regulation with UC model is arachidonic acid (AA), one of the polyunsaturated essential fatty acids reported to exhibit an effective role in chronic inflammation and can enhance immune response [82,83].

## 4. Conclusions

This study demonstrates for the first time the anti-ulcerogenic effect of *A. saligna* in the rat model of acetic-acid induced ulcerative colitis. It explores the anti-inflammatory mechanisms involved as referred from decreased production of inflammatory biomarkers and histological inflammatory cells infiltration. In-line, multivariate data analyses revealed that ASBE nano extract intervention could attenuate the inflammation in UC via restoring normal metabolites level. One of the most appealing metabolites observed via metabolomics analysis is oleamide, suggested being involved in the inflammation process that may give a new therapeutic target for UC through enhancing both phagocytosis and anti-inflammatory activity. More interestingly, the formulation of ASBE on its own as nanoparticles enhanced its anti-ulcer effect and was comparable to the reference drug, dexamethasone.

## Figures and Tables

**Table 1:** Effect of ASBE, nano-extracts, and their combination with nano-Ag on the macroscopic parameters of ulcerative colitis.

Group	Weight/Length Ratio (g/cm)	Lesion Score (0–5)	Ulcer Area (mm^2^)	Ulcer Index (UI)	Mortality (Dead/Total%)
Control Negative	0.127 ± 0.007	0.00 ± 0.00	0.00 ± 0.00	0.00 ± 0.00	0/6 (0.0%)
Control Positive	0.251 ± 0.028 ^a^	5.50 ± 0.879 ^a^	18.75 ± 0.994 ^a^	24.25 ± 1.546 ^a^	1/6 (16.66%)
Dexamethasone	0.155 ± 0.016 *	3.97 ± 0.590 *^a^	11.75 ± 1.258 *^a^	15.72 ± 1.118 *^a^	1/6 (16.66%)
Nano Ag	0.151 ± 0.004 *	4.00 ± 0.182 *^a^	8.87 ± 1.250 *^a^	12.87 ± 1.109 *^a^	0/6 (0.0%)
ASBE	0.202 ± 0.013 *^a^	4.20 ± 0.258 *^a^	13.50 ± 0.577 *^a^	17.70 ± 0.365 *^a^	1/6 (16.66%)
ASBE + Nano Ag	0.174 ± 0.011 *^a^	4.02 ± 0.427 *^a^	8.62 ± 1.109 *^a^	12.65 ± 1.318 *^a^	0/6 (0.0%)
ASB Nano ext.	0.193 ± 0.015 *^a^	4.17 ± 0.550 *^a^	9.95 ± 0.823 *^a^	14.14 ± 0.842 *^a^	1/6 (16.66%)
ASB Nano ext. + Nano Ag	0.146 ± 0.010 *	3.50 ± 0.294 *^a^	8.50 ± 1.291 *^a^	12.00 ± 1.010 *^a^	0/6 (0.0%)

Results are presented as mean ± SD (n = 6). *p* < 0.05 compared to the positive control, one-way ANOVA followed by Tukey test. ^a^ Statistically significant from control negative. * Statistically significant from control positive at *p* < 0.05.

**Table 2:** Metabolites fold change calculated by dividing the mean of each metabolite peak intensity from each of the two groups. Statistical significance was revealed using the non-parametric Mann-Whitney test. Results were considered only significant if *p* ≤ 0.05 using Kruskal-Wallis test analysis.

Metabolites	Fold Change *						
	B/A	C/B	D/B	E/B	F/B	G/B	H/B
Lactic Acid	2.30 *	0.095 *	0.29 *	0.63	0.79	0.46 *	0.80
Oxalic Acid	1.026	0.15 *	1.93	0.42	0.93	0.52	1.15
Pyroglutamic Acid	3.06 *	16.36 *	0.33 *	0.117 *	0.38 *	0.45 *	0.33 *
D-Fructose	2.52*	0.19 *	0.94	0.84	0.98	0.85	1.01
D- Sorbitol	5.77 *	1.027	0.89	0.64	2.46 *	1.47	3.42 *
D-Fructose	21.32 *	0.018 *	0.11 *	0.17 *	0.76	0.31 *	0.48
Arachidonic Acid	0.25 *	7.51 *	3.91 *	2.38	1.44	1.40	1.70
Oleamide	0.051 *	16.84 *	17.53	31.53 *	12.66 *	31.47 *	14.31
Erucylamide	0.23 *	15.90 *	1.99 *	0.92	0.73	0.77	1.49

Statistical comparisons equal the non-parametric Mann-Whitney test was performed. Results were considered only significant if *p* ≤ 0.05 using Kruskal-Wallis test analysis. Sample codes are presented as follows: (A) negative control, (B) positive control, (C) dexamethasone standard, (D) nano-Ag, (E) ASBE, (F) ASBE plus nano-Ag, (G) ASB nano-extract, and (H) ASB nano extract plus nano-Ag. * Denotes metabolites showing significant difference among treated groups.

## Supplementary Materials

The following are available online at https://www.mdpi.com/2079-7737/9/8/195/s1, Figure S1: Representative structures of identified compounds from *n*-BuOH extract of *A. saligna*, Table S1: Gas chromatography-mass spectroscopy (GC-MS) identification of metabolites in rats’ sera based on its RI and MS spectral data.
