# Supplementary material for: Protective Mechanism of Acacia saligna Butanol Extract and Its Nano-Formulations against Ulcerative Colitis in Rats as Revealed via Biochemical and Metabolomic Assays
Source: Biology (Basel). 2020 Jul 30;9(8):195. doi: 10.3390/biology9080195 (PMC7463518; doi:10.3390/biology9080195)
Supplement: Supplementary file 1 [file biology-09-00195-s001.pdf]

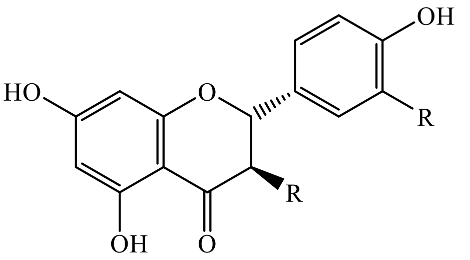

1 R=H, **2** R=OH

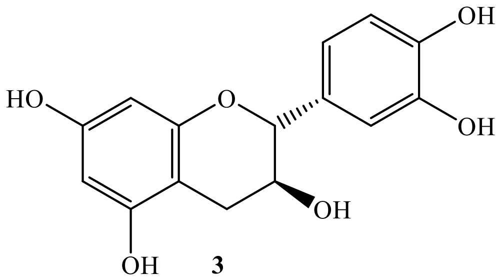

3

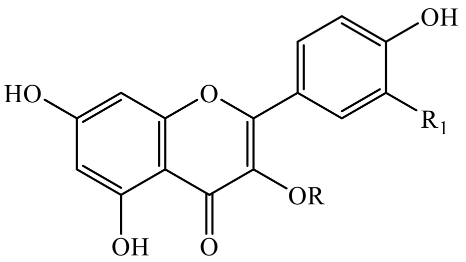

4 R=H, R_1=OH
5 R=Rutinoside, R_1=OH
6 R=R_1=H

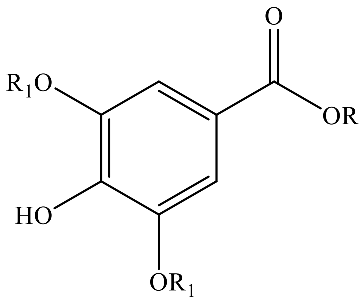

7 R=R_1=H, **8** R=CH_3, R_1=H,
9 R=H, R_1=CH_3

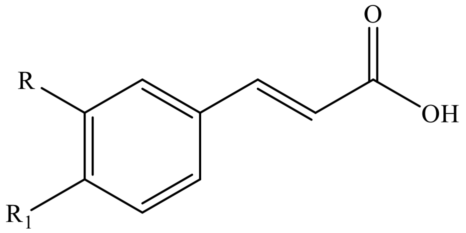

10 R=R_1=H, **11** R=H, R_1=OH,
12 R=OCH_3, R_1=OH, **13** R=R_1=OH

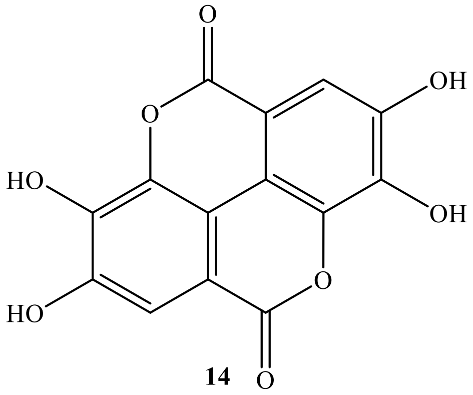

14

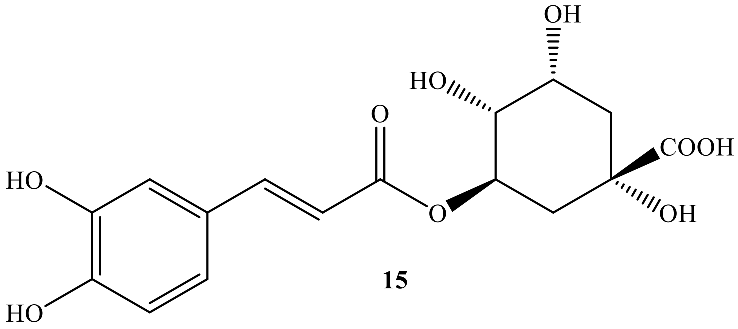

15

Figure S1. Representative structures of identified compounds from *n*-BuOH extract of *A. saligna*.

Table S1. Gas chromatography-mass spectroscopy (GC-MS) identification of metabolites in rats' sera based on RI and MS spectral data.

No	RT[a]	Metabolite	RI[b]	*m/z*	Groups							
					A	B	C	D	E	F	G	H
**1**	**4.644**	**Lactic acid, trimethylsilyl ether, trimethylsilyl ester**	**949**	147	1.02	1.77	0.17	0.52	1.12	1.39	0.81	1.42
2	5.547	Alanine, N-(trimethylsilyl)-, trimethylsilyl ester	975	116	0.02	0.02	0.00	0.02	0.02	0.02	0.01	0.02
3	5.708	Tris(trimethylsilyl)hydroxylamine	981	249	0.02	0.01	0.00	0.01	0.01	0.01	0.01	0.01
4	6.05	α-Hydroxybutyric acid (2TMS)	991	147	10.50	10.16	4.91	7.31	1.45	5.65	2.24	7.41
5	6.486	Oxalic acid, bis(trimethylsilyl) ester	1003	73	17.68	14.33	2.09	27.66	6.06	13.38	7.52	16.51
6	6.879	β-Hydroxybutyric acid (2TMS)	1014	147	0.31	0.14	6.77	0.19	0.25	0.18	0.20	0.19
7	8.142	Valine, N-(trimethylsilyl)-, trimethylsilyl ester,	1051	144	0.01	0.02	0.00	0.01	0.01	0.01	0.01	0.01
8	8.316	2-Ketoisocaproic acid TMS	1056	73	0.82	0.83	0.32	0.89	1.41	1.58	0.98	1.04
9	8.458	Cadaverine tetra-TMS	1060	174	0.01	0.01	0.00	0.01	0.01	0.01	0.00	0.01
10	9.309	Urea, N,N'-bis(trimethylsilyl)-	1085	189	1.13	1.05	0.16	0.07	0.77	1.20	0.50	0.77
11	9.551	Glycerol, tris(trimethylsilyl) ether	1092	147	3.00	0.83	8.09	0.24	0.78	1.22	1.57	0.86
12	9.653	Tris(trimethylsilyl) phosphate	1096	299	4.58	0.37	10.28	0.21	11.88	0.62	0.10	0.25
13	10.088	Isoleucine, N-(trimethylsilyl)-, trimethylsilyl ester, L-	1107	158	0.02	0.02	0.03	0.01	0.01	0.01	0.01	0.02
14	10.243	Proline, 1-(trimethylsilyl)-, trimethylsilyl ester	1111	142	0.02	0.01	0.00	0.01	0.00	0.01	0.01	0.01
15	10.359	Glycine (N,N,O-TMS)	1114	174	0.02	0.03	0.00	0.04	0.05	0.04	0.02	0.02
16	11.025	Glyceric acid, tris(trimethylsilyl) deriv.	1130	147	0.15	0.26	1.00	0.18	0.31	0.24	0.24	0.10
17	13.411	Propanoic acid, 2-oxo-3-(trimethylsilyl)-, trimethylsilyl ester	1189	228	0.02	0.01	0.00	0.01	0.01	0.01	0.00	0.01
18	15.999	Pyroglutamic acid, bis(trimethylsilyl)-	1251	156	0.02	0.07	1.06	0.02	0.01	0.02	0.03	0.02
19	19.16	Fructose-pentakis-O-(trimethylsilyl)-, O-methyloxime	1325	217	25.27	40.49	7.52	38.46	34.05	39.73	34.64	41.01
20	19.64	Xylitol-pentakis-O-(trimethylsilyl)-	1336	217	0.01	0.01	0.01	0.01	0.00	0.00	0.01	0.01
21	20.04	Monoamidomalonic acid, tris(trimethylsilyl)	1346	304	0.01	0.01	0.00	0.00	0.00	0.00	0.00	0.02
22	22.231	Azelaic acid, bis-TMS	1398	317	0.00	0.00	0.00	0.01	0.00	0.00	0.00	0.00
23	22.938	1,5-Anhydro-sorbitol, tetrakis(trimethylsilyl) ether	1415	217	0.07	0.39	0.40	0.35	0.25	0.95	0.57	1.33
24	23.125	Fructosepentakis-O-(trimethylsilyl)-, O-methyloxime	1421	307	1.13	24.19	0.44	2.66	4.12	18.34	7.59	11.68
25	23.637	Glucosepentakis-O-(trimethylsilyl)-, O-methyloxyme,	1433	319	0.04	0.10	14.12	0.04	0.04	0.22	0.06	0.07
26	23.983	Glucose-pentakis-O-(trimethylsilyl)-, O-methyloxyme	1441	205	0.02	0.01	0.02	0.01	0.01	0.01	0.01	0.01

27	25.453	Glucopyranose-pentakis-O-(trimethylsilyl)-	1477	204	0.01	0.01	0.00	0.01	0.01	0.01	0.00	0.01
28	26.283	Mannose-deoxy--tetrakis-O-(trimethylsilyl)	1498	204	0.02	0.02	0.71	0.02	0.04	0.02	0.00	0.01
29	26.388	Galactosepentakis-O-(trimethylsilyl)-, O-methyloxyme	1501	319	0.01	0.01	0.00	0.01	0.00	0.01	0.00	0.01
30	26.767	9-Hexadecenoic acid, trimethylsilyl ester	1510	311	0.01	0.00	0.00	0.01	0.00	0.00	0.00	0.01
31	27.167	Palmitic acid, trimethylsilyl ester	1521	313	0.02	0.02	1.60	0.02	0.02	0.02	0.01	0.03
32	27.231	Myo-Inositol, -hexakis-O-(trimethylsilyl)	1523	305	0.00	0.01	0.00	0.00	0.00	0.00	0.00	0.00
33	27.33	Mannose, 6-deoxy-2,3,4,5-tetrakis-O-(trimethylsilyl)	1525	204	0.02	0.01	0.00	0.01	0.01	0.01	0.01	0.00
34	30.155	Linoleic acid trimethylsilyl ester	1597	337	0.09	0.00	0.00	0.00	0.00	0.01	0.00	0.00
35	30.251	TMS trans-9-octadecenoate	1600	339	0.01	0.00	0.54	0.01	0.01	0.00	0.00	0.00
36	30.733	Stearic acid, trimethylsilyl ester	1613	341	0.02	0.01	0.13	0.02	0.02	0.01	0.01	0.01
37	32.795	Arachidonic acid, TMS	1668	117	0.06	0.02	0.12	0.06	0.04	0.02	0.02	0.03
38	33.143	Oleamide	1678	72	30.09	4.57	19.81	20.62	37.08	14.89	37.01	16.84
39	33.643	Oleamide, trimethylsilyl	1691	131	3.28	0.04	17.27	0.03	0.02	0.02	5.68	0.04
40	35.068	Oleanitrile	1731	122	0.05	0.03	1.18	0.04	0.02	0.02	0.01	0.03
41	36.171	Monopalmitin trimethylsilyl ether	1762	371	0.02	0.00	0.00	0.00	0.01	0.00	0.00	0.00
42	38.6	Monolinoleoylglycerol trimethylsilyl ether	1833	129	0.05	0.05	0.04	0.05	0.06	0.04	0.04	0.04
43	39.572	Erucylamide	1861	126	0.33	0.08	1.20	0.15	0.07	0.06	0.06	0.11
44	43.84	Cholesterol trimethylsilyl ether	1992	368	0.01	0.01	0.00	0.01	0.00	0.01	0.00	0.01
		Total			100.00	100.00	100.00	100.00	100.00	100.00	100.00	100.00

[a] RT: Retention time, [b] RI: retention indices, Groups: **A**) Negative control, **B**) positive control, **C**) dexamethasone, **D**) nano-Ag, **E**) ASBE, **F**) ASBE plus nano-Ag, **G**) ASB nano-extract, **H**) ASB nano extract plus nano-Ag.
